# *Cryptococcus neoformans* and *Cryptococcus gattii* Species Complex Isolates on the Slopes of Mount Etna, SICILY, Italy

**DOI:** 10.3389/fmicb.2019.02390

**Published:** 2019-10-18

**Authors:** Laura Trovato, Salvatore Oliveri, Maria Carmela Esposto, Anna Prigitano, Luisa Romanò, Massimo Cogliati

**Affiliations:** ^1^U.O.C. Laboratory Analysis Unit, A.O.U. “Policlinico-Vittorio Emanuele”, Catania, Italy; ^2^Department of Biomedical and Biotechnological Sciences, University of Catania, Catania, Italy; ^3^Department of Biomedical Sciences for Health, Università degli Studi di Milano, Milan, Italy

**Keywords:** *Cryptococcus neoformans* complex, *Cryptococcus gattii* complex, environment, molecular typing, mating assay

## Abstract

This study investigated the presence of *Cryptococcus neoformans* and *Cryptococcus gattii* species complex isolates on olive trees growing in the Eastern part of Sicily (Italy) characterized by the presence of the volcano Etna and the ability of these fungal pathogens to sexually reproduce on medium containing volcanic soil. Samples from 124 olive trees were collected from 14 different sites around Mount Etna. Eighteen trees (14.5%) resulted colonized by *C. neoformans* VNI-αA isolates, one (0.8%) by VNIV-αD isolates, and two (1.6%) by *C. gattii* VGI-αB isolates. The ability of environmental and reference strains belonging to VNI, VNIV, and VGI molecular types to sexually reproduce on a medium containing volcanic soil was also tested. VNI and VNIV strains were able to produce filaments and basiodiospores more vigorously than on the control medium, whereas VGI strains were not fertile. In conclusion, the present study identified which *C. neoformans* and *C. gattii* species complex genotypes are circulating in Eastern Sicily and confirmed the ecological role of olive trees as environmental reservoir of these pathogens. It also showed that *Cryptococcus* is able to colonize and sexually reproduce in inhospitable environments such as the slopes of a volcano.

## Introduction

Cryptococcosis is a pulmonary or disseminated infection caused by the encapsulated yeasts belonging to *Cryptococcus neoformans* and *Cryptococcus gattii* species complexes. Immunosuppression is the major predisposing condition involved in the development of cryptococcosis, which mainly affects HIV-infected patients, organ transplant recipients, patients with autoimmune diseases and those receiving corticosteroid or immunosuppressive therapies (Rajasingham et al., 2017; Henao-Martínez et al., 2018). Cryptococcosis is acquired from the environment by inhalation of basidiospores or small blastospores that reach the pulmonary alveoli and may frequently disseminate to meninges causing fatal meningitis or meningo-encephalitis (Kwon-Chung et al., 2014). Differences in biology, epidemiology, pathogenicity, clinical manifestations, and drug susceptibility have been observed between the two species complexes (Kwon-Chung and Varma, 2006; Lee et al., 2019). Infections caused by yeasts of the *C. neoformans* species complex, belonging to molecular types VNI, VNII, VNB, VNIII or VNIV, are primarily associated with immunosuppression (Kwon-Chung et al., 2014), whereas some yeasts of the *C. gattii* species complex (molecular types VGI and VGII) frequently infect immunocompetent hosts (Chen et al., 2014). Among each molecular type two mating type alleles can be distinguished, mating type a and mating type a, being the former more frequent than the latter (Heitman et al., 2011).

*C. neoformans* VNI is the most prevalent molecular type worldwide and has been recovered from bird excreta, soil, and trunk hollows of different tree species, suggesting that trees play a major role as reservoir in the environment (Costa et al., 2010; Chowdhary et al., 2012b; Colom et al., 2012; Cattana et al., 2014; Noguera et al., 2015; Cogliati et al., 2016a; Ellabib et al., 2016; Dou et al., 2017). In contrast, *C. neoformans* VNIV is mostly distributed in Europe, and less frequently in North and South America and Japan (Cogliati, 2013). VGI is the prevalent molecular type among the *C. gattii* species complex, which is mainly distributed in tropical and subtropical regions as well as in some geographical area with temperate climate such as the North Pacific coast of North America and Europe (Byrnes and Marr, 2011; Chowdhary et al., 2012a; Hagen et al., 2012; Springer et al., 2014; Cogliati et al., 2016a; Acheson et al., 2018). In addition, particular interest is recently addressed on *C. gattii* VGII which is causing outbreaks in British Columbia (Canada), North Pacific coast of United States, Brazil and Colombia (Lockhart et al., 2016; Acheson et al., 2018; Firacative et al., 2019; Maruyama et al., 2019).

A recent environmental survey carried out in Europe and the Mediterranean area showed the presence of both *C. neoformans* and *C. gattii* species complexes in the environment and that some trees, such as olive trees and carob trees, presented a rate of colonization higher than others (Cogliati et al., 2016a). Furthermore, a niche modeling study considering the European climatic conditions suggested that the potential areas of distribution of the two species complexes is often overlapping and that therefore, in Europe yeasts with different molecular types can frequently interact each with the other (Cogliati et al., 2017).

In Sicily, an island of Southern Italy, *C. neoformans* and *C. gattii* species complex isolates were previously recovered from Messina (Northeast Sicily) in several samples of bird excreta as well as in *Eucalyptus camaldulensis*, *Prunus dulcis* (almond), and *Ceratonia siliqua* (carob) (Criseo and Gallo, 1997; Pernice et al., 1998; Romeo et al., 2012). However, olive trees were not sampled during these environmental surveys. Eastern Sicily is characterized by the presence of Mount Etna, the largest volcano in Europe that reaches an altitude of 3350 m.a.s.l. Volcanic origin of soil, difference in altitudes, and exposure of the different sides of the mountain make unique the biotope of Mount Etna, which is very different from the rest of Sicily characterized by a Mediterranean climate.

The aim of this study, therefore, was to evaluate the distribution and molecular characterization of *C. neoformans* and *C. gattii* species complex isolates in Eastern Sicily, and the ability of these fungal pathogens to sexually reproduce on medium containing volcanic soil.

## Materials and Methods

### Environmental Sampling and Processing

In the present study olive trees growing at different altitude in urban and rural areas around Mount Etna were investigated. The environmental survey was carried out in October–November 2014 and April–May 2015. Samples were collected by rubbing the inner of hollows and fissures of olive trees with a sterile cotton-tipped swab moistened in a solution of sterile distilled water supplemented with chloramphenicol (10 mg/L), and processed and cultured on Niger seed agar as previously reported (Pham et al., 2014; Cogliati et al., 2016a). All brown colonies grown on the plates were isolated and species was identified by microscopy for assessing yeast morphology and capsule presence, and by testing urease activity, ability to grow at 37°C, and to assimilate *myo*-inositol as a carbon source. Isolates belonging to *C. neoformans* or *C. gattii* species complex were differentiated by inoculating a fresh colony onto L-canavanine-glycine-bromothymol blue (CGB) agar prepared as described elsewhere (Shadomy et al., 1987).

### Molecular Typing

Genomic DNA was extracted as described by Viviani et al. (1997). Molecular type and mating type of all isolates was determined by four multiplex PCRs specific for both *C. neoformans* and *C. gattii* species complexes as previously described (Cogliati et al., 2000, 2015; Esposto et al., 2004; Feng et al., 2013). Molecular and mating types were assigned according to the standard nomenclature of the ISHAM Working Group for genotyping of *C. neoformans* and *C. gattii* (Meyer et al., 2009). Strains H99 (VNI-αA), JEC20 (VNIV-**α**D), JEC21 (VNIV-αD), IUM 96–2828 (VNII-**α**A), WM626 (VNII-αA), WM779 (VGIV-αC), NIH312 (VGIII-αB), NIH191 (VGIII-**α**C), WM201 (VGI-αB), and IUM 00–5363 (VGII-**α**B) were used as reference strains. Multi-locus sequence typing (MLST) according to the ISHAM scheme (Meyer et al., 2009) was performed for *C. gattii* species complex isolates. Sequence types (ST) were assigned comparing the MLST profile with those present in the *C. gattii* MLST database^1^.

### Mating Assay

For the mating assay a volcanic soil medium (VSM) was prepared suspending 50 g of Etna volcanic soil in 1 L of distilled water. The suspension was boiled for at least 30 min and then filtered through gauze. Volume was adjusted with further distilled water and 20 g of agar were added. The medium was sterilized by autoclave and then poured in 90-mm Petri dishes. For sugar volcanic soil medium (SVSM), 0.5 g of glucose were added. Each plate containing VSM and SVSM was inoculated with a mix of two strains of opposite mating type as well as with the two strains independently to exclude haploid fruiting. Cultures were incubated at 25°C in the dark for at least 3–4 weeks and checked once a week for production of hyphae and basidiospores. One plate containing the control medium Murashige and Skoog agar (Sigma-Aldrich, Milano, Italy) was also inoculated as described above in order to test if the two strains were actually able to mate. Mating assays were performed as reported in Table 1. JEC20 (VNIV-αD), JEC21 (VNIV-αD), SPCCON11HO1-1 (VNIV-αA), and SPCCON11HO1-2 (VNI-αA) were used as reference tester strains, and strain ITBRF221HO1-5 (VGI-αB), isolated from Southern Italy (Montagna et al., 2018), was used for the mating assays with the VGI isolates from Sicily.

**TABLE 1:** Results of environmental samplings performed on the olive trees growing around Mount Etna.

**Town**	**Etna side**	**Altitude (m.a.s.l.)**	**No. of sampled olive trees**	**No. of samples**	**Positive trees**	**Positive samples**	**Species/variety**	**Molecular type/mating type**
Riposto	East	7	4	5	1	1	*C. neoformans* var. *grubii*	VNI/aA
Giarre	East	81	12	12	–	–	–	–
Nunziata	East	201	8	10	1	1	*C. neoformans* var. *grubii*	VNI/aA
Piedimonte Etneo	East	348	10	12	4	4	*C. gattii* species complex	VGI/αB
							*C. neoformans* var. *grubii*	VNI/αA
							*C. neoformans* var. *grubii*	VNI/αA
							*C. neoformans* var. *grubii*	VNI/αA
Zafferana Etnea	East	574	12	12	–	–	–	–
S. Venera	East	656	13	15	1	1	*C. neoformans* var. *grubii*	VNI/αA
Acireale	Southeast	161	9	10	2	2	*C. neoformans* var. *grubii*	VNI/αA
							*C. neoformans* var. *grubii*	VNI/αA
Catania	Southeast	7	8	9	4	5	*C. neoformans* var. *grubii*	VNI/αA
							*C. neoformans* var. *grubii*	VNI/αA
							*C. neoformans* var. *grubii*	VNI/αA
							*C. neoformans* var. *grubii*	VNI/αA
							*C. neoformans* var. *grubii*	VNI/αA
Ragalna	Southwest	830	15	53	3	4	*C. neoformans* var. *neoformans*	VNIV/αD
							*C. neoformans* var. *grubii*	VNI/αA
							*C. neoformans* var. *grubii*	VNI/αA
							*C. gattii* species complex	VGI/αB
Adrano	Southwest	584	13	13	1	1	*C. neoformans* var. *grubii*	VNI/αA
Paternò	Southwest	300	2	2	–	–	–	–
S.M. Licodia	Southwest	442	2	3	–	–	–	–
Mompilieri	Southwest	680	2	2	–	–	–	–
Mojo Alcantara	North	535	14	14	4	4	*C. neoformans* var. *grubii*	VNI/αA
							*C. neoformans* var. *grubii*	VNI/αA
							*C. neoformans* var. *grubii*	VNI/αA
							*C. neoformans* var. *grubii*	VNI/αA

### Statistics

Data were analyzed using the MedCalc Statistical Software version 17.9.2 (MedCalc Software bvba, Ostend, Belgium; 2017)^2^. Percentage of positive samples from North, East, Southeast, Southwest sides of Mount Etna as well as comparison of positive samples at different altitude were compared using the two-tailed χ^2^ test.

## Results

**FIGURE 1:**
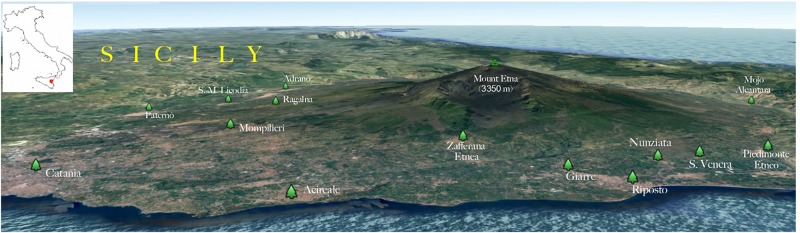
Three-dimensional map showing the geographical distribution of the environmental sampling sites. The insert shows where the geographical area is located in Italy (red area).

**FIGURE 2:**
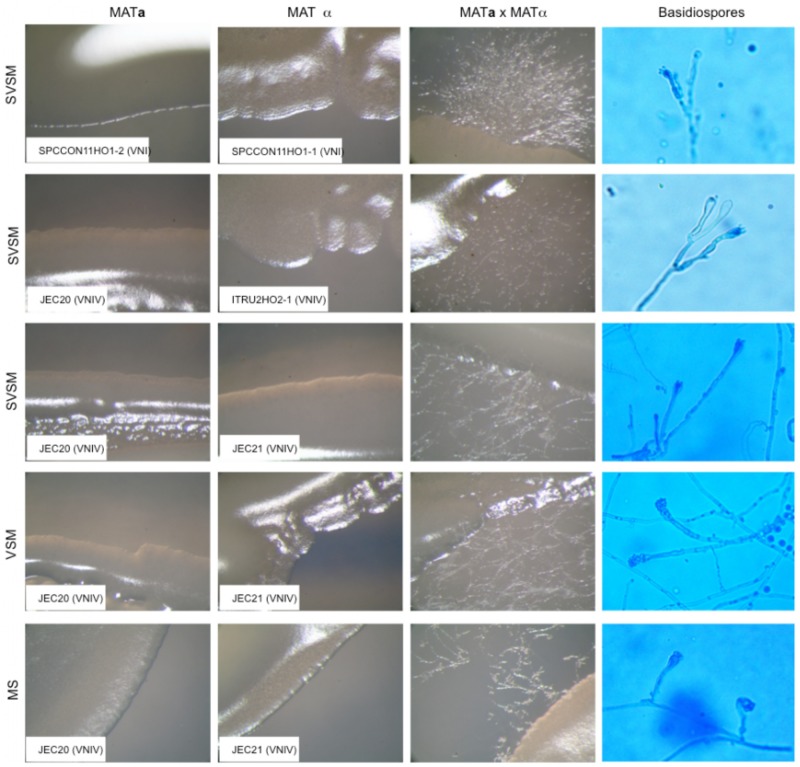
Results of fertile mating type assays. MATa, MATα, and MATa × MATα cultures were observed at 50× magnification with a stereomicroscope (Zeiss, Essingen, Germany). Basidiospores were observed at 400× magnification with a light microscope Axioskope 40 (Zeiss). SVSM = Sugar volcanic soil medium; VSM = Volcanic soil medium; MS = Murashige and Skoog agar.

The geographical distribution of environmental samplings is shown in Figure 1 and the results are reported in Table 1. One hundred and seventy-two samples were collected from 124 olive trees. A total of 21 isolates from 19 olive trees (15.3%) were identified as *C. neoformans* species complex whereas only two trees (1.6%), one growing on the East and the other on the Southwest side of Mount Etna, were colonized by *C. gattii* species complex isolates. Fourteen olive trees (14 samples) were sampled on the North, 59 (66 samples) on the East, 17 (19 samples) on the Southeast, and 34 (73 samples) on the Southwest side of Mount Etna, and the rate of positive trees was 28.6% (4/14), 11.9% (7/59), 35.3% (6/17), and 11.8% (4/34), respectively. Statistical analysis showed no significant difference between the four sides of Mount Etna, although the percentage of positive trees was higher in the North and the Southeast sides despite the number of specimens sampled was lower. Considering the altitude threshold value of 400 m.a.s.l., statistical analysis showed no significant difference of percentage of positive trees at different altitude, although it was higher at altitudes below 400 meters (22.6%, 12/53 vs. 12.7%, 9/71). Among the 21 isolates of the *C. neoformans* species complex, twenty were *C. neoformans* var. *grubii* belonging to molecular type VNI, mating type aA and one was *C. neoformans* var. *neoformans*, VNIV, mating type aD. The remaining two isolates were *C. gattii*, VGI, mating type aB. MLST analysis showed that both *C. gattii* VGI isolates belonged to ST197 which matched with the ST of other four isolates present in the MLST database: CCA320 and CCA321 environmental isolates from Barcelona (Spain), and CBS6290 and IHEM11792 clinical isolates from Kinshasa (Congo Republic). Results of mating assays showed that all tested strains were able to grow on both VSM and SVSM, although more vigorously on the latter one. The mating assay with the two VNIV reference tester strains (JEC21 × JEC20) produced filaments and basiodiospores on both VSM and SVSM, which showed to be a more suitable media compared to Murashige and Skoog agar (Figure 2). In addition, sporulation was also observed for the mating between the environmental VNIV isolate from Sicily (ITRU2HO2-1) and JEC20 (Figure 2). Among mating assays performed with VNI isolates only the one between the two tester strains (SPCCON11HO1-1 × SPCCON11HO1-2) resulted fertile on SVSM (Figure 2). None of the *C. gattii* mating assays resulted fertile after 4 weeks of incubation (Table 2).

**TABLE 2:** Mating assays performed in the present study.

**Mating assay**		**Days of incubation to produce filaments**		
	
**Mating type α strain**	**Mating type a strain**	**Volcanic soil medium**	**Volcanic soil medium + glucose**	**Murashige and Skoog agar**
**Cryptococcus neoformans VNI**				
SPCCON11HO1-1 (tester strain)	SPCCON11HO1-2 (tester strain)	NF	12	NF
ITRU3HO1-1 (strain from Etna)	SPCCON11HO1-2 (tester strain)	NF	NF	NF
**Cryptococcus neoformans VNIV**				
JEC21 (tester strain)	JEC20 (tester strain)	4	4	4
ITRU2HO2-1 (strain from Etna)	JEC20 (tester strain)	NF	9	NF
**Cryptococcus gattii VGI**				
ITPDM3HO1-1 (strain from Etna)	ITBRF221HO1-5 (strain from Southern Italy)	NF	NF	NF
ITRU1HO1-1 (strain from Etna)	ITBRF221HO1-5 (strain from Southern Italy)	NF	NF	

## Discussion

This study confirms the presence of *C. neoformans* and *C. gattii* species complex on olive trees growing in Eastern Sicily on the four sides of Mount Etna with a high prevalence on North and Southeast sides. The percentage of positive olive trees was 15.3 and 1.6% for *C. neoformans* and *C. gattii* species complex, respectively, confirming the important role of these trees as an ecological niche for both species complexes. A recent environmental survey carried out in Apulia (another region of Southern Italy) reported a percentage of positive olive trees (18%) similar to that found in our study (Montagna et al., 2018). Since Sicily is the third Italian region for the production of olive oil, with more than 150,000 hectares of its territory (about 6%) dedicated to olive tree cultivation, this region represents an area with high suitability for the survival of *Cryptococcus* as also confirmed by a recent niche modeling study (Cogliati et al., 2017). VNI was the prevalent molecular type among isolates collected in the present study as observed in other regions of Southern Italy (Romeo et al., 2012; Cogliati et al., 2016a; Montagna et al., 2018), confirming the ubiquitous presence of this fungal pathogen. The unique isolate with molecular type VNIV was found in an olive tree on the Southwest side of Etna and in particular at an altitude of 820 m.a.s.l. This could reflect the ability of VNIV isolates to tolerate lower temperature better than VNI and *C. gattii* VGI isolates as previously reported (Cogliati et al., 2017). Other authors reported the isolation of this yeast in the nostrils of squirrels living in the Appenine area of Calabria (Iatta et al., 2015). All together these data confirm that VNIV isolates are able to survive in the European areas with a sub-continental climate (Cogliati et al., 2017). Both *C. gattii* species complex isolates belonged to VGI molecular type and ST197 genotype. This genotype was previously reported to be genetically related to several other European genotypes forming a Mediterranean cluster (Hagen et al., 2012). Therefore, the isolates recovered in Sicily during this study are probably endemic of the Mediterranean area and have been present in the environment since long time.

The mating type a was the only mating type identified among all isolates. Although mating type **a** isolates were not recovered during this study, future surveys could be able to detect them considering that such isolates have been already found in Southern Italy (Viviani et al., 2001; Cogliati et al., 2016a; Montagna et al., 2018). Mating assays showed that *C. neoformans* and *C. gattii* species complex strains are able to grow on media containing volcanic soil with and without addition of glucose. This means that volcanic soil is a suitable substrate for the growth of these fungi and therefore, that the peculiar characteristics of the soil on Mount Etna do not limit their survival. In addition, our results showed that *C. neoformans* species complex strains belonging to VNI and VNIV molecular types are able to sexually reproduce on medium containing volcanic soil, and that one environmental VNIV isolate recovered from Mount Etna was fertile. This is in agreement with the recent findings reporting that VNIV population is more recombinant than VNI population and thus sexual reproduction occurs more frequently in the former than in the latter (Cogliati et al., 2016b, 2019). In contrast, none of the tested *C. gattii* strains was able to mate probably due to the fact that VGI strains are the least fertile strains among *C. gattii* species complex and the absence of adequate tester strains (Springer et al., 2017). Several studies report the isolation of *C. neoformans* species complex strains from soil samples (Machado et al., 1993; Montagna et al., 2003, 2018; Cogliati, 2013; Escandón and Castañeda, 2015; Cogliati et al., 2016a) and, recently, it was also showed its ability to mate and produce basidiospores on media prepared with different soil substrates (Cogliati, 2018). However, at present, similar studies on media containing volcanic soil have not yet been carried out. The ability of this yeast to grow on the volcanic soil confirms that *C. neoformans* species complex strains are able to colonize a wide range of microhabitats including those more inhospitable. Volcanic soil of Mount Etna is a sub-acid substrate (pH 6.8), rich of microelements such as iron and copper, with a medium content of potassium, phosphorus and magnesium, but poor of nitrogen and calcium (D’Antone et al., 2017). These characteristics are very similar to those recommended to induce filamentation and sporulation of cryptococcal yeasts (Hsueh et al., 2011) and therefore, volcanic soil of Mount Etna results a suitable substrate for sexual reproduction of this fungal pathogens. Furthermore, the ability of cryptococcal yeasts to grow and produce blastospores and basidiospores on the volcanic soil represents a potential source of infection since soil aerosols could transfer small cells and spores in pulmonary alveoli of humans and animals causing the onset of the infection. In conclusion, this study identified some of *C. neoformans* and *C. gattii* species complex genotypes present in Eastern Sicily and confirmed the ecological role of olive trees as environmental reservoir of these pathogens. It also showed that *Cryptococcus* is able to colonize and sexually reproduce in inhospitable environments such as the slopes of a volcano.

## Data Availability Statement

All datasets generated for this study are included in the manuscript/supplementary files.

## Author Contributions

LT, SO, and MC designed the study. LT and SO performed the environmental samplings. MC, ME, AP, and LR performed the molecular typing and mating assay. LT, SO, and MC analyzed the data. LT and MC wrote the manuscript. All authors read and approved the final version of the manuscript.

## Conflict of Interest

The authors declare that the research was conducted in the absence of any commercial or financial relationships that could be construed as a potential conflict of interest.

## Footnotes

1 www.mycologylab.org

2 http://www.medcalc.org
